# The Impact of Physical Activity at School on Body Fat Content in School-Aged Children

**DOI:** 10.3390/ijerph191912514

**Published:** 2022-09-30

**Authors:** Katarzyna Ługowska, Wojciech Kolanowski

**Affiliations:** 1 Faculty of Medical and Health Sciences, Siedlce University, 08-110 Siedlce, Poland; 2 Faculty of Health Sciences, Medical University of Lublin, 20-400 Lublin, Poland

**Keywords:** children, fat mass, obesity, physical activity

## Abstract

(1) Background: Excessive amounts of adipose tissue is a health risk. The aim of this study was to assess the impact of increased physical activity (PA) at school on body fat content in children aged 10 to 12 years over a 2-year follow-up. (2) Methods: Children born in 2007 (n = 245) in two groups, (1) standard PA and (2) increased PA at school, 4 and 10 h of physical education lessons per week, respectively. BIA measurements of body fat content were taken twice a year. Results were interpreted based on children’s fat content reference curves. (3) Results: During 2 years of observation, the percentage of children with excessive fat mass (overweight and obese) increased by one-third (from 28.11% to 39.67%) in the group of standard PA, while decreased by one-third in the increased PA one (from 28.92% to 21.00%); with normal fat content increased by one-quarter in the increased PA group (from 59.86% to 76.26%) and decreased by one-tenth in the standard PA one (from 61.61% to 56.29%). (4) Conclusions: An increase in PA at school has a positive impact on children’s body fat content. It is recommended to increase the number of physical education lessons at school, which has a positive effect on children’s health, reducing the risk of obesity.

## 1. Introduction

Overweight and obesity among school-aged children increases are one of the most serious public health problems [1,2,3]. It is estimated that excessive body weight affects more than 340 million children worldwide, with the prevalence in children in high-income countries [4,5,6]. The World Health Organization (WHO) indicates that one in three children has excessive body weight. It is predicted that more than 60% of children with excessive body weight will be overweight or obese in adulthood [7,8]. In Poland, almost one-third of school-aged children have excessive body weight (overweight or obese) [9,10,11].

The WHO defines obesity as an excessive accumulation of adipose tissue that poses a health risk [12]. Body mass index (BMI) is a commonly used method to assess body weight; however, in children, the interpretation of BMI is difficult due to rapid growth and development, as well as differences in body structure [13,14]. That is why the direct measurement of body fat mass seems to be more suitable to determine the degree of obesity [15,16,17].

The measurement of body composition, including the amount of body fat mass, is most commonly carried out using the BIA (Bioelectric Impedance Analysis) [18,19,20]. The BIA measures impedance, or electrical resistance, which consists of the resistance and reactance of soft tissues through which a low-intensity electric current is passed. The BIA is useful for the determination of the ratio between body fat mass and lean body mass, not only in different patient groups but also in healthy individuals (e.g., monitoring the effectiveness of diet or sports training) [21].

Inter-population variation, lifestyle, level of physical activity (PA), body structure, ethnicity, age, and gender may account for the observed differences in body fat level [21]. During puberty, sex hormones induce a distinct sexual dimorphism: boys gain proportionally more muscles and lean tissue, while girls store adipose tissue as a natural part of the development of female body shape [20,22]. In girls, adipose tissue is mainly distributed in the lower half of the body (hips, buttocks), and its mass increases with age until the end of puberty. Boys are characterized by a more central type of adiposity (mainly the upper half of the body: neck, shoulders, lower abdomen) [23]. However, a sedentary lifestyle and unhealthy diet lead to excessive fat mass and obesity. Excessive adiposity is the main risk factor for most common lifestyle diseases, such as hypertension, ischemic heart disease, hyperlipidemia, type 2 diabetes, and many types of cancers, and it disturbs the hormonal balance. Increased body fat mass poses a serious health risk, affecting physiological functions and performances [24,25]. There is a confirmed relationship between a sedentary lifestyle and increased adipose tissue content in both boys and girls [26]. Low PA is not only associated with an increase in adipose tissue content but also results in a decrease in relative muscle mass, which is consequently associated with lower physical fitness [27]. It should be noted that maintaining normal muscle mass requires regular PA [28,29]. However, only a small percentage of school-aged children and adolescents meet the requirements of the daily PA level [30]. The WHO recommends that children and adolescents should perform at least 60 min of moderate- to high-intensity PA each day [31]. In Poland, before the COVID-19 pandemic, only 17% of children and adolescents were undertaking moderate PA [32]. Increased PA in children leads to a reduced risk of obesity and improves body composition, health, and fitness [33]. It is estimated that, on average, 80% of school-aged children do not reach the recommended PA level [34]. Through compulsory PE lessons and additional organized PE classes, the school enables children to fulfill the recommendations regarding PA [35,36,37]. Moreover, participation in organized PA, especially team games, can bring benefits beyond physical health [32,34,38,39,40,41,42,43].

Counteracting overweight and obesity in children and adolescents requires an equally increased PA and the limitation of a sedentary lifestyle, as well as a healthy diet [4,31,44]. These are basic conditions for maintaining good health from childhood to old age [1,4,9].

Reference percentile grids for body fat mass in children and adolescents in many countries have not been developed. Therefore, in most studies, fat mass in school-aged children is interpreted in relation to McCarthy et al. (2006) body fat reference curves [22,45]. The aim of this study was to assess the impact of increased physical activity at school on body fat content in children aged 10 to 12 years over a 2-year follow-up.

## 2. Materials and Methods

### 2.1. Participants

At the testing time, all participants were free of acute injuries and did not report any current musculoskeletal system pain. The sample size was justified by a priori power analysis in G*power software (Version 3.1.9.7; Universität Kiel, Germany) [46] with a type I error rate of 0.05 and 80% statistical power. Overall, the analysis indicated that 122 participants SC and 123 GC (total 245) are sufficient to observe significant large-sized acute effects (Cohen’s d = 0.80). 

A total of 304 children born in the year 2007 were examined, of whom 161 (75 girls; 86 boys) attended GC and 143 (67 girls; 76 boys) attended SC. During the study, approximately 23% of children from GC (n = 38) and approximately 15% of children from SC (n =21) were excluded from the final analysis. The exclusion criteria were as follows: absence at one of the measurement sessions, failure in being promoted to the next grade, the lack of the child’s consent for the measurement, diagnosed chronic diseases, or having a pacemaker of the heart that could have affected the BIA measurement result [47,48,49]. However, none of the children’s parents reported problems with diagnosed chronic diseases. Moreover, according to the adopted criteria, sick leave on the day of the measurements, trauma, and wounds were among the factors excluding them from further analysis. All participants were free from acute trauma at the time of measurements and did not report any current pain in the musculoskeletal system [47,48,49]. The children were not part of integrated units, which might have included children with health disorders affecting the final results. In cases where a child was admitted to SC or GC class during the study as a result of a transfer from another class or another school, these results were excluded from the final analysis.

The date of the planned measurements was agreed upon with the teachers and the school management. One month before the measurement, the parents received written information about the date of the measurement with a request to prepare the child for it (described in detail in Section 2.2). Along with their consent to participate in the study, the parents declared that they would prepare the child for measurements in accordance with the specified requirements. Other studies showed a positive effect of involving parents in preparation for the measurements [50].

The study was conducted in six primary schools in Siedlce (Poland), a typical, medium-sized city in central Poland, which had parallel classes with a standard and elevated number of PE lessons, so-called general education classes (GC) and sports classes (SC), respectively.

In Poland, education with an elevated number of PE lessons (SC) in regular primary schools began with the fourth grade of school. The mandatory number of PE lessons for GC and SC children was four and ten hours per week, respectively. PE lessons consisted of organized PA in the form of conditioning exercises, sports forms of movement, and team games [37].

The main study was preceded by a pilot study conducted among children aged approximately 10 years (grade 4) from May to June 2017 in GC and SC classes n = 43 (GC 22; SC 21). The aim of the pilot study was to test the methods adopted and evaluate the procedures and the flow of measurements. The methods were planned properly and were applied smoothly. The requirements for conducting the pilot study were similar to others of this type [45]. The methods previously verified in the pilot study were used in the main study.

The interpretation of the results focused on analyzing the variability of the fat content category according to the class profile, reflecting the number of PE lessons and the gender and age of the children. The results were systematically analyzed and made available to parents during meetings once in a semester.

### 2.2. Procedure

This study was an observational controlled cohort follow-up with a control group that involved the assessment of body fat mass among school-aged children attending classes of different levels of PA over a period of 2 years. The test group was children of elevated PA at school (SC), and the control group was children of standard PA at school (GC). The study was conducted on the same group of children from September 2017 to October 2019. At the start of the study, the children were approximately 10 years old and were entering the fourth grade of school. The study was completed when the children were approximately 12 years old and were attending the sixth grade. During the study, the participants were divided by gender and class profile. The children and their parents gave informed consent to participate in the study. Each participant was informed about the objective of the study and the confidential nature of the results. The study was approved by the Research Ethics Committee at Siedlce University (No. 2/2016), and a positive opinion was expressed by the school management, tutors, teachers, parents, and children.

The study was conducted by a team of five licensed dietitians. Prior to the study’s start, the team members were trained in the use of a BIA body composition analyzer, data coding, and the confidentiality of the results. The study team members knowingly and voluntarily agreed to participate in the study. Measurements were taken in the presence of teachers at 5–6 month intervals. The first (initial) measurement session was carried out from 1 to 30 September 2017, the second from 1 to 30 March 2018, the third from 1 to 31 September 2018, the fourth from 1 to 30 March 2019, and the fifth (final) from 1 to 31 September 2019. The adopted way of interpreting the measurement sessions in this study was as follows: I-September 2017; II-March 2018; III-September 2018; IV-March 2019; V-September 2019. A total of five measurement sessions were conducted, and the results were systematically processed and analyzed. Anthropometric measurements were carried out in accordance with the methodology described in the previous works [42,50].

Parents were asked to stop the child from eating copious meals after 9 p.m. in the evening before the measurement day and on the measurement day to have a light breakfast. Just before the measurement, the children were asked not to eat or drink until the measurement was completed. In addition, they were asked to refrain from significant physical exertion 24 h before the measurement, and the last activity should be performed at least 12 h before it. The parents received the above-mentioned guidelines in paper form 7 days before the planned measurement and a reminder message from teachers 4 days before [47,48,49].

### 2.3. Fat Content Measurement

Body fat content was assessed using a Tanita SC-240 MA (Tanita Cooperation, Tokio, Japonia) body composition analyzer device having the CE0122 EU certificate [45]. According to the specification, the device is designed to measure individuals aged 5–99 [45,46,47,49]. Before starting the measurements, the research team made sure that the equipment was in a stable place, the tests were always carried out between 10 a.m. and 11 a.m. standing and observing all necessary measurement guidelines. [49].

Measurements were taken by members of the study team in classrooms, divided by gender and class profile. The tests were carried out according to a standard protocol in accordance with the manufacturer’s recommendations without shoes or socks, with clean and dried feet, and wearing light clothing. The children were asked to (1) avoid exertion and excessive fluid intake before the test; (2) were properly prepared by their parents (point 2.2); (3) at least 30 min before the measurement, children were asked to empty their intestines and bladder; (4) the children were standing for at least 5 min before the measurement to redistribute the tissue fluids; (5) measurement was performed while standing [49,50,51,52,53].

Prior to each measurement, the analyzer surfaces were disinfected. Then, after each surface had dried, the participants were instructed to stand up straight on the four electrodes with bare feet, arms away from the torso, and legs slightly apart [48,49,50,54]. The above procedure was implemented uniformly in all schools throughout the entire study period.

The percentage of fat mass (FM%) suggested by McCarthy [22] was noted. The body fat mass was interpreted on the basis of the WHO-approved reference curves, and the range of the broad normal limit was assessed from the 9 to the 75 percentile [22]. Percentiles 2 and 85 indicated children with deficient and excess adipose tissue, respectively, whereas ≥95 percentile indicated obese children.

### 2.4. Statistical Analysis

Statistical calculations were performed using the Microsoft Excel spreadsheet (Microsoft, Intentional Software, Washington, DC, USA) [55] and the Statistica 13 Programme (Stat Soft, Cracow, Poland) [55]. The level of statistical significance was assumed that the level α ≤ 0.05. Mean, median, standard deviations, and 95% confidence intervals were calculated at the level of class, gender, and age profile.

The following tests were performed: the Shapiro–Wilk test to assess the normality, a paired sample *t*-test to was used to compare children’s body fat at the class profile and gender, and an analysis of the fat content variability between the initial and final measurements. One-way ANOVA was used to determine the differences between the percentage of the fat mass category variables of the SC and GC. Effect sizes (ESs) were calculated utilizing Cohen’s d. The threshold values for ES statistics were: >0.2 small, >0.5 moderate, >0.8 large, >1.3, very large [56].

## 3. Results

### 3.1. Group Characteristics

Finally, results from 245 children (48% girls—G; 52% boys—B) were included in the study. The number of children in both groups was similar (SC *n* = 122; GC *n* = 123). In the increased PA group (SC), 143 children (G 67; B 76) were included in the study. However, due to not meeting the criteria, approximately 15% of the participants (n = 21) (G 9%, B 20%) were not included in further analysis. The final analysis included 122 children from the SC group, who attended all the measurement sessions (G 61; B 61). From the GC group, 161 children were included in the study (G 75; B 86). The final analysis included 123 GC children who attended all the measurement sessions (G 57; B 66). Changing school or class (13%) and joining a class during the study (10.5%) were the most common factors excluding them from further procedures.

### 3.2. Body Fat Content

The body fat content in the children ranged from 5.70 to 48.90% (GC 5.70–48.90%; SC 3.00–39.70%). Higher values were observed in the GC group (Table 1). On average, children from the GC group had slightly higher body fat content at the start of the study (GC 20.25%; SC 19.30%; *p* = 0.390) as well as at the end of the study (GC 20.38%; SC 19.55%; *p* = 0.550).

The body fat content in the GC girls ranged from 5.90 to 39.70%, whereas in the SC girls, from 5.50 to 39.70% (Table 1). Girls from the SC group had a lower fat content compared to GC ones (SC 21.59%, GC 22.56%; *p* = 0.050). There was a noticeable decrease in the average body fat content in the GC and SC girls after the first year of follow-up, followed by a gradual increase. In girls with normal body weight, the body fat content was close to 20% (KO 19.57%; KS 19.01%; *p* = 0.010). In underweight girls, it was approximately 10.05% (KO 10.81%; KS 9.29%; *p* = 0.002). In overweight girls it was 27.51% (KO 29.98%; KS 25.04%; *p* = 0.000), and in obese ones, 35% (KO 35.67%; KS 34.26%; *p* = 0.010). The differences were statistically significant (*p* = 0.050).

The body fat content in the GC boys ranged from 5.70 to 48.90% and from 3.00 to 36.10% in the SC ones (Table 1). On average, throughout the study period, the SC boys had a lower fat content compared to the GC ones (16.31% and 17.84%, respectively; *p* = 0.002). Overall, girls had a higher fat content compared to boys (22.7% and 17.07%, respectively; *p* = 0.000). In normal body weight boys, the body fat content was on average 14.83% (GC 15.08%; SC 14.59%; *p* = 0.050). In underweight boys, it amounted to 9.42% (KO 9.56%; KS 9.28%; *p* = 0.020). In overweight boys, it was 23.52% (KO 24.27%; KS 22.78%; *p* = 0.002), and in obese ones, 31.29% (KO 33.42%; KS 29.16%; *p* = 0.000). The differences were statistically significant (*p* = 0.030).

### 3.3. Categorization of Body Fat Content

**Figure 1:**
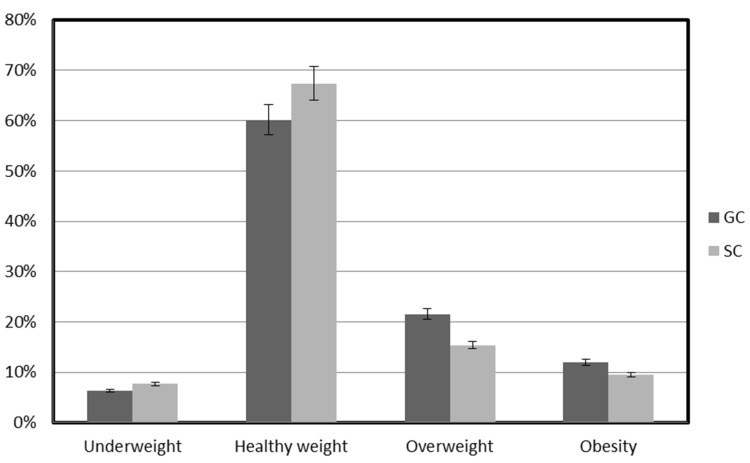
Categorization of body fat content in GC and SC children during the study period. GC—general education classes (standard PA, 4 h a week); SC—sport classes (elevated PA, 10 h).

Although the mean body fat content did not indicate great differences between GC and SC, the interpretation of the body fat content based on the reference curves showed much greater differences. Throughout the study period, body fat content related to normal body weight was found in 64.04% of children (GC 60.14%; SC 67.95%; *p* = 0.000). Underweight was noted in nearly 7% of the children (GC 6.34%; SC 7.62%; *p* = 0.001). Excessive body weight was found in nearly 29% of the children (GC 33.49%, SC 24,32%; *p* = 0.002), of which overweight concerned 18.39% of the children (GC 21.54%; SC 15.25%; *p* = 0.003), and obesity, 10.57% of children (GC 11.95%; SC 9.17%; *p* = 0.000) (Figure 1). Excessive body fat content (overweight and obesity) was more frequently identified in GC children than in SC ones.

The highest mean percentage of underweight children was noted at the beginning of the study (grade 4, mean age 10.27 years) and concerned on average 10.75% of the children (GC 10.28%; SC 11.22%; *p* = 0.756) (Table 2). The lowest one occurred at the end of the study and concerned on average 3.39% of children (GC 4.04%; SC 2.75%; *p* = 0.039). The highest mean percentage of normal body fat content was found in the children of grade 6 (mean age 12.26 years) and concerned on average 66.27% of the children (GC 56.29%; SC 76.25%; *p* = 0.047). The lowest one was noted in grade 4 (mean age 10.27 years) and concerned on average 60.73% of the children (GC 61.61%; SC 59.86%; *p* = 0.953). The highest mean percentage of overweight children was noted at the end of the study (grade 6, mean age 12.26 years) and concerned on average 20.59% of the children (GC 26.65%; SC 14.53%; *p* = 0.017). The lowest one was noted at the beginning of the study (grade 4, mean age 10.90 years) and concerned on average 17.37% of the children (GC 20.78%; SC 13.96%; *p* = 0.020). The highest mean percentage of obese children occurred in grade 4 (mean age 10.90 years) and concerned on average 11.71% of the children (GC 11.52%; SC 11.91%; *p* = 0.400). The lowest one was noted at the end of the study (grade 6. mean age 12.26 years) and concerned on average 9.75% of the children (CG 13.02%, SC 6.47%; *p* = 0.026).

Throughout the entire study period, normal body fat content was found on average in 62.46% of the girls (GC—59.29%; SC—65.64%; *p* = 0.000); underweight in 7.17% of the girls (GC—6.09%, SC—8.25%, *p* = 0.010); overweight in 19.43% of the girls (GC—22.78%; SC—16.08%; *p* = 0.000); and obesity in 10.93% of the girls (GC—11.84%; SC—10.00%; *p* = 0.500) (Table 3). Normal body fat content was more common in SC girls than in GC ones (*p* = 0.010), while overweight and obesity were more common in GC ones (*p* = 0.020).

The normal body fat content in the GC girls amounted to, on average, 21.12% (7.76 kg) at the first measurement session and 21.71% (10.24 kg) at the last measurement session (an increase of 0.56%, *p* = 0.006). The normal body fat content in the SC girls was higher at the first measurement session compared to the GC ones and amounted to, on average, 21.38% (8.03 kg), while it was lower at the last measurement session—21.68% (9.21 kg) (an increase of 0.30%, *p* = 0.001). Statistically significant differences were observed for normal-weight girls between the initial and final measurement sessions (*p* = 0.000).

The mean body fat content in the underweight GC girls was on average 11.41% (3.35 kg) at the first measurement session and 13.42% (5.35 kg) at the final one (an increase of 2.01%, *p* = 0.000), while in the SC girls, it was 11.14% (3.35 kg) at the initial and 13.10% (5.15 kg) at the final measurement sessions (an increase of 1.96%, *p* = 0.020).

In the GC girls with excessive fat content (overweight and obese), the average fat content amounted to 31.54% (14.79 kg) at the initial measurement session and 32.53% (19.72 kg) at the final one (an increase of 0.99%; *p* = 0.003). In the SC girls, these values were 31.82% (15.25 kg) and 32.45% (18.45 kg), respectively (an increase of 0.63%; *p* = 0.020). Significant differences were found between the fat contents of the overweight SC girls between the initial and final measurement sessions (*p* = 0.000), while in the GC ones, the differences were not significant (*p* = 0.260).

Throughout the entire study period, normal body fat content was noted on average in 65.62% of the boys (GC 60.92%; SC 70.25%; *p* = 0.000); underweight in 6.79% (GC—6.60%, SC—6.99%, *p* = 0.050); overweight in 17.36% (GC 20.31%; SC 14.42%; *p* = 0.000); and obesity in 10.21% (GC 12.09%; SC 8.33%; *p* = 0.000) (Table 4). Normal body fat content was noted more often in the SC boys than in the GC ones (*p* = 0.000), while overweight and obesity were more often in the GC than the SC ones (*p* = 0.000).

The normal body fat content in the GC boys amounted to, on average, 16.82% (6.34 kg) at the initial measurement session and 17.48% (9.02 kg) at the final one (an increase of 0.66%, *p* = 0.005). The normal body fat content in the SC boys was higher at the initial measurement session (amounted to, on average, 17.36%, 6.64 kg) and lower at the final one (17.10%; 8.14 kg) (a decrease of 0.26%, *p* = 0.003). Statistically significant differences were observed for normal-weight boys between the initial and final measurement sessions (*p* = 0.000).

The mean body fat content of the underweight GC boys was, on average, 10.77% (3.28 kg) at the beginning of the study and 12.17% (4.03 kg) at the end (an increase of 1.40%, *p* = 0.010). In the underweight SC boys, body fat content was on average 11.50% (3.57 kg) at the initial measurement session and 10.38% (4.17 kg) at the final one (a decrease of 1.12%, *p* = 0.000).

In the excessive body fat content GC boys (overweight and obese), the mean body fat content amounted to 30.40% (17.21 kg) at the initial measurement session and 31.83% (20.65 kg) at the final one (an increase of 1.43%; *p* = 0.520). In the SC ones, these values were 27.46% (13.28 kg) and 27.22% (12.80 kg), respectively (a decrease of 0.31%; *p* = 0.000). Significant differences were demonstrated between the body fat content in overweight SC boys between the initial and final measurement sessions (*p* = 0.000), while in the GC ones, the differences were not significant (*p* = 0.260).

Normal body fat content was found on average in 62.46% of the girls and 65.62% of the boys, and underweight was more often identified in girls than boys (B 6.79%; G 7.17%; *p* = 0.050). Overweight concerned 19.42% of the girls and 17.36% of the boys (*p* = 0.000), and obesity—10.93% and 10.21%, respectively (*p* = 0.050).

The body fat content in the children increased correspondingly with age regardless of the class profile, while differences in the fat content between the GC and SC groups and between genders were evident. The average body fat content in the children amounted to 19.60% (GC 20.19%; SC 19.00%; *p* = 0.000), the lowest body fat content in children was noted in grade 4 (initial stage) with 19.04%, and the highest was in grade 6 (final stage)—19.96% (*p* = 0.010).

Taking into account the entire study period, normal body fat content was found on average in 60.14% of the GC children and 67.95% of the SC ones (*p* = 0.000); a high percentage of children with excessive body fat content (overweight and obesity) was reported. Obesity and overweight were more common in the GC than the SC children (GC 33.51%; SC 24.42%; *p* = 0.000), while underweight was more common in the SC ones (GC 6.34%; SC 7.62%; *p* = 0020). The girls, especially the GC ones, were characterized by higher body fat content compared to the boys. There was an increase in the body fat content in the girls throughout the entire study period, while the boys had a lower body fat content at the final stage compared to the initial measurement (grade 4).

### 3.4. Analysis of Fat Content Variability during the Study

**Figure 2:**
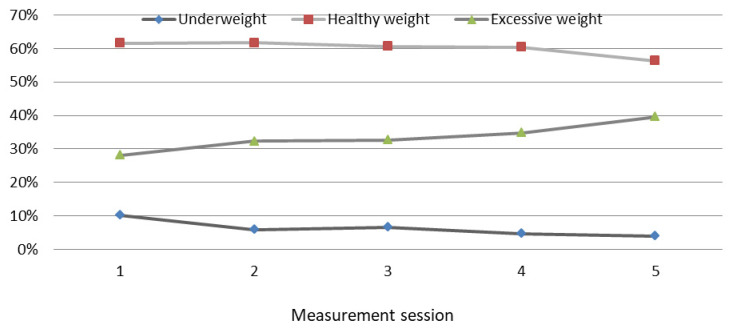
Changes in the body fat content category in GC children during the course of the study. Excessive weight—overweight and obesity. Changes in the fat mass % category in GC children during the course of the study.

During the course of the study, an increasing share of children with excessive body fat content (overweight and obesity) was observed in the GC group, from an initial 28.11% to 39.67% at the final measurement session (*p* = 0.007) (Figure 2). A decreasing share of underweight children was shown, from 10.28% to 4.04% (*p* = 0.001). The percentage of GC children with normal body fat content decreased from 61.61% to 56.29% (*p =* 0.000) (Figure 2). The highest percentage of underweight GC children was shown at the beginning of the study (10.28%) and the lowest at the fifth measurement session (54.04%; mean age of 12.26 years). The share of children with excessive body fat increased with age, while normal body fat content decreased. The highest percentage of GC children with normal body fat content was shown at the beginning of the study (61.61%) and the lowest one was at the final measurement session (56.29%; *p* = 0.003).

**Figure 3:**
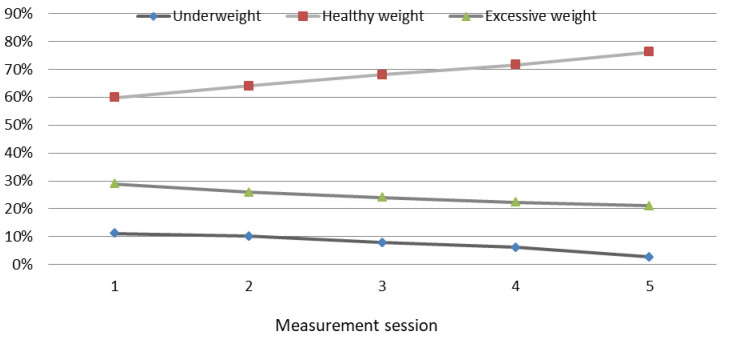
Changes in the body fat content categories in SC children during the course of the study. Excessive weight—overweight and obesity. Changes in the fat mass % categories in SC children during the course of the study.

In contrast to the GC children, in the SC ones, a decrease in the percentage of children with excessive body fat content was shown, from an initial 28.92% to 21% at the final measurement session (*p* = 0.001). The share of children with body fat content deficiency (underweight) decreased from 11.22% to 2.75% (*p* = 0.000) (Figure 3). During the course of the study, the share of SC children with normal body fat content increased from 58.96% to 76.25% (an increase of 17.29%) (*p* = 0.000). The highest percentage of SC children with body fat deficiency (underweight) was found at the beginning of the study (11.22%) and the lowest at the final measurement session (2.75%). The share of children with excessive body fat content (overweight and obese) was the highest at the initial measurement session (28.92%) and the lowest at the final measurement session (21%; *p* = 0.003).

**Figure 4:**
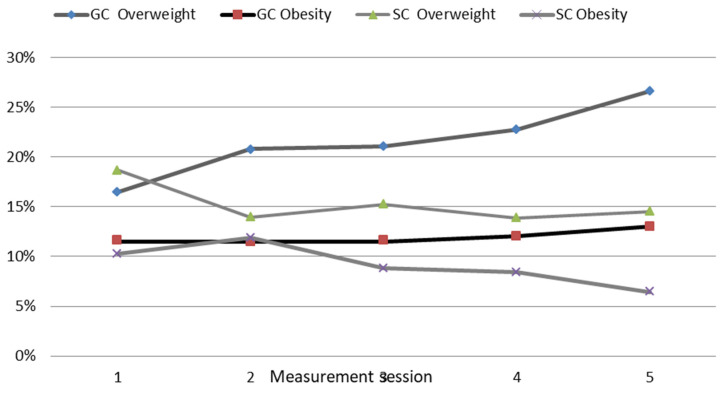
A comparison of the number of overweight and obese GC and SC children during the course of the study. GC—general education classes (standard PA, 4 h a week); SC—sport classes (elevated PA, 10 h). A comparison of the percentage of overweight and obese GC and SC children during the course of the study. Excessive weight—overweight and obesity; GC—general education classes (standard PA, 4 h a week); SC—sport classes (elevated PA, 10 h a week).

Compared to the initial stage of the study, there was an increase in overweight GC children of 10.15% (from 16.50% to 26.65%; *p* = 0.000) and increase in obese children of 1.41% (from 11.61% to 13.02%; *p* = 0.050) (Figure 4). In the SC ones, a significant decrease in overweight children (from 18.67% to 14.53%; *p* = 0.000) and a decrease in obese children of 3.78% (from 10.25% to 6.47% *p* = 0.020) were shown. Overall, there was a clear downward trend in the share of overweight and obese children in the SC group and a significant upward trend in excessive body fat content in the GC group during the course of the study.

In general, the study showed an increase in the share of GC boys and girls with excessive body fat content and a decrease in the SC group (Figure 4). At the beginning of the study, 17.58% of the children were overweight, including 16.50% in the GC group and 18.67% in the SC one (*p* = 0.350). At the end of the study, almost 21% of the children were overweight, including 26.65% in the GC group and 14.53% in the SC group (*p* = 0.000). At the beginning of the study (grade four), approximately 61% of the children had normal body fat content (GC 61.61%; SC 59.86%; *p* = 0.520). In contrast, the final measurement session (grade six) showed a higher percentage of children with normal fat content in the SC group (76.25%) and a lower one in the GC group (56.29%) *p* = 0.000.

### 3.5. Analysis of Fat Content Variability between Initial and Final Measurements

**Figure 5:**
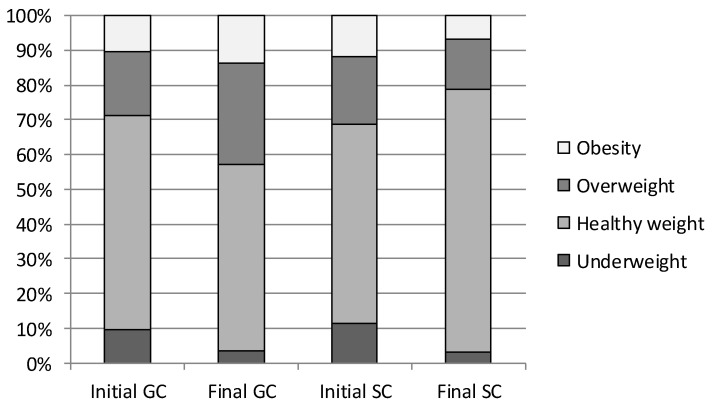
Percentage of GC and SC girls in particular fat mass % categories—comparison between initial (I) and final (F) measurement session. Initial—initial measurement session; Final—final measurement session; GC—general education classes (standard PA, 4 h a week); SC—sport classes (elevated PA, 10 h). I—Initial measurement; F—final measurement; GC—general education classes (standard PA, 4 h a week); SC—sport classes (elevated PA, 10 h a week).

On average, almost 63% of the girls had normal body fat content throughout the entire study period, more often in the SC group (GC 59.26%; SC 65.64%; *p* = 0.000) (Figure 5). Overweight was noted in 22.77% of the GC girls and 16.08% of the SC ones (*p* = 0.000). Obesity affected 11.84% of the GC girls and 10.02% the SC ones (*p* = 0.030). The percentage of the SC girls with normal body fat content increased from 57.05% to 75.59% (*p* = 0.000); the percentage of the overweight ones decreased from 19.45% to 14.47% (*p* = 0.003). However, in GC, the opposite trend was observed, i.e., a decrease in the normal body fat content girls from 61.62% to 53.71% (*p* = 0.008) and an increase in the overweight ones from 18.45% to 29.25% (*p* = 0.001) and the obese ones from 10.35% to 13.52% (*p* = 0.320). In both the SC and GC girls, a decrease in body fat deficiency (underweight) was observed during the course of the study (*p* = 0.005) (Figure 5).

**Figure 6:**
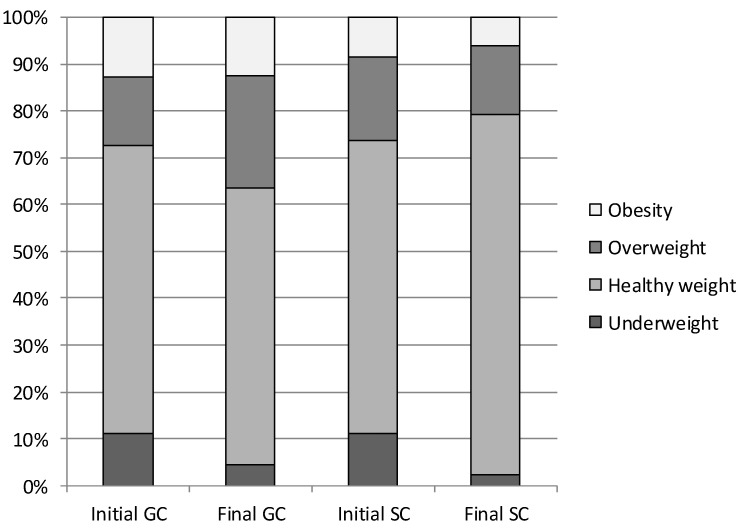
Percentage of GC and SC boys in particular body fat content categories—comparison between initial and final measurement session. Initial—initial measurement session; Final—final measurement session; GC—general education classes (standard PA, 4 h a week); SC—sport classes (elevated PA, 10 h).

On average, almost 65.62% of the boys had normal body fat content throughout the entire study period, with a higher percentage in the SC group than in the GC one (GC—60.99%; SC—70.25%; *p* = 0.000) (Figure 6). Underweight was present in 6.60% of the GC boys and 6.99% of the SC ones (*p* = 0.520). During the study, a decrease in the percentage of overweight SC boys from 17.89% to 14.59% (*p* = 0.050) and an increase in the boys with normal body fat content from 62.66% to 76.91% (*p* = 0.004) were found. In the GC group, the opposite trend was observed, i.e., an increase in overweight boys from 14.55% to 24.05% (*p* = 0.000) and a decrease in boys with normal body fat content from 61.60% to 58.88% (*p* = 0.004). Obesity decreased in the SC group by one-quarter (initial 8.45%, final 6.06%) and remained at the same level in the GC group (initial 12.87%, final 12.52%).

## 4. Discussion

An unhealthy lifestyle (including an unhealthy diet and low PA) causes adipose tissue gain and contributes to an increased risk of obesity and related chronic diseases even at a young age. All types of interventions involving increased PA are appropriate strategies to reduce the risk of excessive adipose tissue content [57,58,59]. In the present study, an association was observed between the duration of PA at school in the form of PE lessons and the incidence of excessive adipose tissue content. The significant difference in the percentage of body fat content that was observed between children of different levels of PA was also confirmed by other studies [60,61,62,63,64].

It was noted that the adipose tissue content increased correspondingly with the age of children, irrespective of the class profile, while differences in the GC and SC groups and between the genders were evident. The average body fat content was 19.57% (GC 20.19%; SC 18.95%), with the lowest value observed in grade 4—19.40% and the highest in grade 6—19.96%. Children from the GC group had higher body fat content at the beginning (GC 20.25%; SC 19.30%) and at the end of the study (GC 20.38%; SC 19.55%). These observations are in line with other studies [65,66,67]. In the study of Vehrs et al. (2022), the observed body fat content in 12-year-old boys and girls was higher compared to this study (21.60% vs. 16.74% and 24.60% vs. 23.18%, respectively) [65]. In the study of Santos et al. (2019), girls had a higher body fat content, while boys presented a higher fat-free mass content [66]. Additionally, girls had a higher fat content compared to boys (G 22.07%; B 17.07%). These data are consistent with the previous studies that suggested that gender differences in adipose tissue content existed well before the puberty period [68]. There are gender differences in the timing and size of regional fat distribution [69]. The increase in adipose tissue content in adolescent girls is primarily associated with the formation of the female body shape [69].

On average, normal body fat content was present in 64% of the children (GC 60.14%; SC 67.95%), underweight in approximately 7% (GC 6.35%; SC 7.62%), overweight in 18.40% (GC 21.54%; SC 15.25%) and obesity in 10.75% of children (GC 11.96%; SC 9.17%). This study showed that overweight and obesity interpreted on the basis of reference curves of body fat content were more frequently found among older children (12 years old), while underweight was more often observed among younger children (10 years old). The prevalence of overweight and obesity is consistent with previous studies [69,70]. For example, Mascherini et al. (2019) assessed the prevalence of body fat content in sport-active children [71]. Compared to the cited study, in this study, a higher percentage of overweight children (16.20% vs. 18.40%) and a similar percentage of obese children (10.57% vs. 10.80%) were found [71]. An earlier study by Etchison et al. (2011) concerned with young sport-active children showed a lower obesity percentage compared to our study (9% vs. 10.75%) [72].

Too low body fat content was observed similarly in girls than boys (on average 7.17% vs. 6.79%). In the study by Vehrs et al. (2022), boys were more often underweight [65]. Kalnin et al. (2015) showed a higher proportion of underweight children than in this study [73].

Overweight and obesity are most often diagnosed on the basis of BMI values. However, many researchers confirm that BMI should not be the only one parameter for determining the presence of overweight or obesity, as it is not closely correlated with adipose tissue content [74]. A normal BMI value and high adipose tissue content are increasingly common and increase health risks [42]. Therefore, assessing the relationship between BMI and the percentage of adipose tissue content is important in the diagnosis of overweight and obesity [22]. The results of the body fat content measured in this study compared to the previously reported BMI in the same group of children showed some differences [42]. On average, during the entire study period, overweight according to BMI and body fat content reference curves in the GC children was 18.90% and 21.50%, respectively, while in the SC children, it was 17.08% and 15.25%, respectively [42]. Next, obesity in the GC group concerned 9.19% and 11.97%, and in the SC group—8.36% and 9.17%, respectively. Additionally, in the study by Petri et al. (2020) differences between the body fat content and BMI were demonstrated [75]. According to BMI, normal weight was presented in 78.0%, overweight in 18.7%, and obesity in 3.3% of the individuals, and according to body fat content, it was 75.0%, 14.0%, and 11.0%, respectively [75]. Other studies also showed an increase in the prevalence of obesity and overweight based on body fat content compared to BMI values in the same group. For example, in the study by Mascherini et al. (2019), it was demonstrated that the prevalence of obesity based on body fat content increased significantly in both girls and boys; the prevalence of obesity increased from 3–4% to about 10% in both genders [71]. The presented results are in line with other studies showing that girls had higher adipose tissue content than boys [76,77,78]. Earlier reports, such as Sardinh et al. (1999) suggested that the triceps skinfold gave the best result to determine the relationship between BMI and percentage of body fat content in healthy boys and girls aged 10 to 15 years, while BMI and arm circumference were considered as a reasonable second choice [79]. However, these parameters should be used with caution in the assessment of individuals doing sports [80]. Nevertheless, the BIA method offers more precise body fat measurement.

Increasing PA in school with more PE lessons can be an effective and promising way to promote a healthy lifestyle and encourage children to spend time actively. The results of this study suggest that the participation of children in additional organized forms of PA at school may bring the desired health effect [35,36]. This was also indicated by other authors such as Grancher et al. (2017) or Casolo et al. (2019) [81,82]. Strengthening efforts to promote PA and a healthy diet in children and adolescents is essential to improve the health status of future adult populations.

The study presented here has some limitations. The study included only 2 years of follow-up. It was planned to continue the study with the same group of children until they graduated from primary school (grade 8, 15 years). However, the outbreak of the COVID-19 pandemic and the associated frequent lockdowns and remote learning caused the study to be discontinued. Furthermore, the study was limited to only a relatively small sample of children aged between 10 and 12 years. It is worth extending the research among children in other age groups and from larger areas. Another limitation is that adipose tissue content is affected not only by the PA level but also by dietary behavior and socio-economic status; however, this analysis did not take these into account. The level of PA outside school was not taken into account, and no questions were asked about the form of spending free time. The regularity of participation in PE lessons was also not analyzed. The nutritional behavior of children and the influence of families and peers on food preferences are also important. These aspects are not covered in this paper.

A strength of the study was the selection of groups of children with increased and standard physical activity at school (10 and 4 h of PE per week, respectively), with very similar numbers of participants (122 and 123, respectively). Another strong point was the study team, whose members were properly qualified to conduct and interpret the study. The team used the same EU-certified equipment with software allowing for a very accurate analysis. This excluded the risk of errors in this area. In addition, only children who attended all the measurement sessions were included in the analysis, which increased the accuracy of the results. A final strength of the study was the regular meetings with parents, at which the study team communicated the results to parents and helped interpret them. Obesity in children is a public health problem that is increasing worldwide. Participation in organized PA during school age should be strongly recommended as it brings many health benefits for children. 

## 5. Conclusions

During 2 years of follow-up, the percentage of children with excessive body fat content (overweight and obese) increased by one-third (from 28.11% to 39.67%) in standard PA children, while it decreased by almost one-third in increased PA children (from 28.92% to 21.00%). The percentage of children with normal body fat content increased by one-quarter in the increased PA group (from 59.86% to 76.25%), and slightly decreased by one-tenth in the standard PA group (from 61.61% to 56.29%). Girls, especially in the standard PA group, were more likely to have excessive body fat content compared to boys. An increase in the PA level at school by a two and a half times increase in the number of PE lessons (from 4 to 10 h a week) positively impacted body fat content in children. It is recommended to increase the number of PE lessons in primary school, which has a positive effect on children’s body weight and body fat content. This can help to prevent the development of overweight and obesity among children and teenagers.

## Figures and Tables

**Table 1:** Body fat content in GC and SC children, percentage (%), and weight units (kg).

Measurement Session	Average Age (Years)	Fat Mass % (kg)													*p*	ESs
		Mean Total	GC						SC							
			Mean	Median	Min.	Max.	SD	95% CI	Mean	Median	Min.	Max.	SD	95% CI		
Average																
I	10.27	19.77 ** (8.10)	20.25 (8.44)	18.10 (6.09)	8.30 (1.80)	47.40 (40.60)	7.90	7.02–9.03	19.30 (7.76)	18.20 (6.45)	6.50 (1.70)	37.30 (21.10)	7.39	6.51–8.38	0.390	0.300
II	10.90	19.04 (8.28)	19.79 (8.86)	18.00 (7.30)	5.70 (1.60)	46.50 (41.80)	8.09	7.19–9.25	18.30 (7.70)	16.90 (6.35)	6.40 (1.70)	38.00 (21.90)	7.15	6.35–8.18	0.170	0.900
III	11.27	19.55 (8.93)	20.41 (9.62)	18.60 (7.80)	6.50 (1.60)	49.00 (45.40)	8.13	7.22–9.29	18.69 (8.24)	17.05 (7.10)	3.00 (1.30)	39.00 (22.90)	7.44	6.61–8.51	0.110	0.050
IV	11.90	19.53 (9.66)	20.15 (10.25)	19.10 (8.50)	7.50 (2.30)	45.90 (50.00)	8.22	7.31–9.40	18.91 (9.08)	17.85 (7.40)	5.70 (1.70)	38.90 (27.70)	7.73	6.87–8.84	0.300	0.245
V	12.26	19.96 (10.45)	20.38 (10.93)	19.00 (9.40)	5.90 (2.20)	48.90 (56.50)	8.24	7.32–9.42	19.55 (9.97)	18.65 (8.20)	6.00 (1.80)	39.70 (26.70)	8.00	7.11–9.16	0.550	0.520
Girls																
I	10.27	21.77 ** (8.73)	22.12 (8.71)	21.90 (8.00)	8.30 (1.80)	34.34 (18.10)	6.76	5.71–8.30	21.38 (8.76)	20.50 (7.50)	6.50 (1.70)	37.30 (21.10)	8.16	6.93–9.94	0.590	0.600
II	10.90	21.18 (9.02)	21.80 (9.26)	21.30 (8.40)	6.70 (2.20)	37.30 (21.20)	7.25	6.12–8.90	20.57 (8.79)	18.70 (7.50)	6.40 (1.70)	38.00 (21.90)	7.75	6.58–9.44	0.370	0.060
III	11.27	21.84 (9.77)	22.40 (10.00)	21.50 (8.50)	6.50 (1.60)	38.10 (23.20)	6.94	5.86–5.15	21.29 (9.54)	19.90 (8.10)	5.50 (1.50)	39.00 (22.90)	7.76	6.45–9.26	0.410	0.390
IV	11.90	22.39 (10.95)	22.88 (11.21)	23.10 (10.00)	8.30 (2.30)	39.20 (26.80)	7.36	6.22–9.03	21.90 (10.70)	20.00 (8.90)	6.50 (1.80)	38.90 (27.70)	7.97	6.76–9.70	0.490	0.210
V	12.26	23.18 (12.00)	23.56 (12.20)	23.90 (11.00)	5.90 (2.20)	39.00 (28.40)	7.50	6.33–9.20	22.81 (11.80)	21.40 (10.00)	6.00 (1.80)	39.70 (26.70)	8.01	6.80–9.75	0.590	0.420
Boys																
I	10.27	17.80 ** (7.46)	18.39 (8.17)	15.70 (5.85)	8.4 (2.20)	47.4 (40.60)	8.44	7.20–10.19	17.22 (6.76)	15.50 (5.60)	9.2 (2.40)	34.3 (18.60)	5.70	4.86–6.98	0.360	0.420
0.42II	10.90	16.91 (7.54)	17.78 (8.46)	15.55 (6.00)	5.7 (1.60)	46.5 (41.80)	8.35	7.13–10.09	16.04 (6.62)	14.90 (5.50)	7.9 (2.20)	36.1 (18.40)	5.72	4.85–6.96	0.170	0.220
III	11.27	17.26 (8.09)	18.4 (9.24)	16.15 (6.90)	7.2 (2.10)	49.0 (45.50)	8.67	7.40–10.46	16.10 (6.94)	14.20 (5.20)	3.0 (1.30)	33.90 (17.90)	6.33	5.37–7.71	0.080	0.485
IV	11.90	16.67 (8.38)	17.42 (9.30)	16.20 (7.40)	7.5 (2.00)	45.9 (50.00)	8.14	6.95–9.82	15.92 (7.47)	14.90 (6.00)	5.7 (1.70)	32.00 (19.20)	6.23	5.28–7.58	0.240	0.256
V	12.26	16.74 (8.90)	17.21 (9.67)	16.40 (7.75)	6.7 (2.20)	48.9 (56.50)	7.75	6.62–9.36	16.28 (8.14)	14.40 (6.40)	6.1 (1.90)	33.40 (23.90)	6.59	5.59–8.02	0.470	0.450

GC—general education classes (standard PA, 4 h a week); SC—sport classes (elevated PA, 10 h a week); ** fat mass in percentage; SD—standard deviation; 95% CI-Confidence Interval; *p*—*t*-test; ESs— Effect sizes; Min.—minimum; Max.—maximum; measurement sessions: I—September 2017; II—March 2018; III—September 2018; IV—March 2019; V—September 2019.

**Table 2:** The percentage of fat mass categories in the GC and SC children during the course of the study.

Fat Mass Category	Measurement Session																			
	I				II				III				IV				V			
	Average Age																			
	10.27				10.90				11.27				11.90				12.26			
	The Type of the Class																			
	GC	SC	F	*p*	GC	SC	F	*p*	GC	SC	F	*p*	GC	SC	F	*p*	GC	SC	F	*p*
Underweight	10.28	11.22	0.096	0.756	5.95	10.11	0.622	0.432	6.66	7.85	0.367	0.546	4.80	6.17	0.505	0.479	4.04	2.75	4.413	0.039
Healthy weight	61.61	59.86	0.003	0.953	61.75	64.02	0.071	0.789	60.66	68.07	1.017	0.314	60.40	71.76	0.026	0.870	56.29	76.25	7.270	0.047
Overweight	16.50	18.67	0.764	0.390	20.78	13.96	3.031	0.020	21.04	15.23	0.111	0.741	22.75	13.87	0.182	0.672	26.65	14.53	5.248	0.017
Obesity	11.61	10.25	0.731	0.400	11.52	11.91	0.028	0.867	11.64	8.85	0.785	0.386	12.05	8.40	0.282	0.601	13.02	6.47	3.395	0.026

GC—general education classes (standard PA, 4 h a week); SC—sport classes (elevated PA, 10 h); p-ANOVA; Measurement session: I—September 2017; II—March 2018; III—September 2018; IV—March 2019; V—-September 2019.

**Table 3:** The percentage of fat mass categories in the GC and SC girls during the course of the study.

Variables	Measurement Session																			
	I				II				III				IV				V			
Average age (years)	10.27				10.90				11.27				11.90				12.26			
Class type	GC	SC	F	*p*	GC	SC	F	*p*	GC	SC	F	*p*	GC	SC	F	*p*	GC	SC	F	*p*
Underweight	9.58	11.45	0.708	0.078	6.02	11.45	0.001	0.990	6.78	8.45	0.114	0.738	4.55	6.87	0.038	0.846	3.52	3.05	0.080	0.090
Healthy weight	61.62	57.05	0.268	0.606	62.23	59.11	0.472	0.494	59.19	66.43	1.976	0.164	59.72	70.04	0.024	0.876	53.71	75.59	3.025	0.033
Overweight	18.45	19.45	0.010	0.249	20.50	15.87	0.212	0.651	22.53	15.87	6.180	0.020	23.15	14.74	0.250	0.155	29.25	14.47	2.031	0.031
Obesity	10.35	12.05	0.060	0.2139	11.25	13.57	0.377	0.552	11.50	9.25	0.382	0.553	12.58	8.35	3.159	0.028	13.52	6.89	3.014	0.025

GC—general education classes (standard PA, 4 h a week); SC—sport classes (elevated PA, 10 h); p-ANOVA; Measurement session: I—September 2017; II—March 2018; III—September 2018; IV—March 2019; V—September 2019.

**Table 4:** The percentage of fat mass categories in the GC and SC boys during the course of the study.

Variables	Measurement Session																			
	I				II				III				IV				V			
Average age (years)	10.27				10.90				11.27				11.90				12.26			
Class type	GC	SC	F	*p*	GC	SC	F	*p*	GC	SC	F	*p*	GC	SC	F	*p*	GC	SC	F	*p*
Underweight	10.98	11.00	0.277	0.104	5.89	8.78	0.936	0.338	6.55	7.25	0.002	0.980	5.05	5.47	0.665	0.110	4.55	2.45	0.313	0.578
Healthy weight	61.60	62.66	0.487	0.489	61.28	68.92	0.181	0.671	62.12	69.71	0.115	0.734	61.08	73.08	0.316	0.576	58.88	76.91	5.232	0.031
Overweight	14.55	17.89	0.003	0.984	21.05	12.05	0.042	0.839	19.55	14.59	0.002	0.960	22.35	13.00	3.145	0.23	24.05	14.59	0.261	0.616
Obesity	12.87	8.45	2.004	0.025	11.78	10.25	0.051	0.826	11.78	8.45	1.987	0.192	11.52	8.45	0.208	0.308	12.52	6.05	3.929	0.067

GC—general education classes (standard PA, 4 h a week); SC—sport classes (elevated PA, 10 h); p-ANOVA; Measurement session: I—September 2017; II—March 2018; III—September 2018; IV—March 2019; V—September 2019.

## Data Availability

Data are available upon request.
